# Hyperphagia severity is underestimated in adults with Bardet-Biedl syndrome – a mixed-method cross-sectional study in the United Kingdom

**DOI:** 10.3389/fendo.2026.1858350

**Published:** 2026-07-02

**Authors:** Jean Mossman, Sarah Flack, Elise Gamertsfelder, Nicolas Touchot, Eric Low, Philip L. Beales

**Affiliations:** 1 Department of Health Policy, Medical Technology Research Group, London School of Economics, London, United Kingdom; 2 Great Ormond Street Hospital for Children, London, United Kingdom; 3 Eric Low Consulting Ltd, East Lothian, United Kingdom; 4 Rhythm Pharmaceuticals, Inc., Boston, MA, United States; 5 Genetics and Genomic Medicine Programme, University College London, Great Ormond Street Institute of Child Health, London, United Kingdom

**Keywords:** appetite regulation, Bardet-Biedl syndrome, hyperphagia, melanocortin-4 receptor (MC4R) pathway, severity scales

## Abstract

**Introduction:**

Bardet-Biedl syndrome (BBS) is a rare genetic disease caused by primary ciliary dysfunction leading to retinal degeneration, rod–cone dystrophy, polydactyly, hyperphagia, early‐onset obesity, renal dysfunction, hypogonadism, and learning difficulties. Hyperphagia, a pathological insatiable hunger leading to excessive and unusual food intake and persistent, obsessive food-seeking behaviours, is a frequent symptom associated with BBS. This study assessed hyperphagia severity in adults with BBS living in the UK, using a standard structured questionnaire accompanied by an in-depth semi-structured interview.

**Methods:**

This non-interventional, cross-sectional study included 51 adults with BBS (49.0% males; 56.9% aged 18–34 years; mean [SD] body mass index [) BMI] of 36.5 [8.8] kg/m^2^ [range 21.8–59.6]). Participants were excluded if they were currently prescribed or had previously taken setmelanotide. Most were of White ethnicity (82.4%), and age at diagnosis ranged from birth to 50 years. Participants completed a 14-item hyperphagia questionnaire comprising multiple-choice and open-ended questions; a subset (n=15) participated in semi-structured interviews. Interview transcripts were coded systematically by a qualitative researcher and reviewed with a BBS clinical expert to assign severity ratings. Semi-structured interviews were designed to address known limitations of self-reporting in this population, including the disability paradox, stigma, and cognitive difficulties.

**Results:**

Questionnaire data classified hyperphagia as severe in 5.9% (3/51) of participants, moderate in 45.1% (23/51), and mild in 49.0% (25/51), whereas semi-structured interviews classified hyperphagia as severe in 66.7% (10/15) participants, moderate in 26.7% (4/15), and mild in 6.7% (1/15). Notably, 86.7% (13/15) participants were assigned a higher severity level via interviews than questionnaires, while only two participants had the same classification across both methods. In a sub-analysis of participants with BMI ≥30 kg/m^2^ (n=39), semi-structured interviews classified hyperphagia as severe in 69.2% (9/13) and moderate in 30.8% (4/13); none were classified as mild hyperphagia.

**Conclusions:**

This study reveals a high prevalence of severe hyperphagia in adults with BBS based on semi-structured interviews and highlights substantial underreporting of hyperphagia severity when relying on self-reported questionnaires alone. The findings support incorporating semi-structured interviews as a mixed-method approach to provide a more comprehensive assessment of hyperphagia burden in adults with BBS.

## 1 Introduction

Bardet-Biedl syndrome (BBS) is a heterogeneous, pleiotropic genetic disease associated with variants in at least 26 genes and four modifier genes identified to date (1). BBS presents with a broad clinical spectrum of symptoms (1–3), including retinal degeneration, rod–cone dystrophy, polydactyly, hyperphagia, early‐onset obesity, renal dysfunction, hypogonadism, and learning difficulties. Although the published literature typically reports BBS as being diagnosed in approximately 1 in 100, 000 to 160, 000 individuals in North America and Europe (4–6), with higher rates observed in certain genetically isolated populations (7, 8), clinical experts believe the true prevalence may be greater than these estimates.

The melanocortin-4 receptor (MC4R) pathway regulates appetite, energy expenditure, and satiety (9, 10). Disruption of this pathway in BBS leads to reductions in, or loss of, the satiety signal, which may lead to hyperphagia (11, 12). Hyperphagia is characterised by a pathological insatiable hunger leading to excessive and unusual food intake and persistent, obsessive food-seeking behaviours, which in turn may cause early-onset obesity (1, 10).

In BBS, early-onset obesity is typically unresponsive to diet and lifestyle modifications due to its neurological and behavioural underpinnings (1), with both hyperphagia and obesity having substantial and broad effects on health and quality of life (1, 13–15). Patients and families report negative impacts of hyperphagia on quality of life, relationships, productivity, school performance, and emotions (13, 14, 16, 17). Obesity affects physical health, since it is strongly associated with the development of diseases or metabolic complications such as type 2 diabetes and cardiovascular, endocrine, renal, and liver diseases (18). Obesity is also associated with negative impacts on mental health, often resulting from the experience of stigma towards the patient and caregivers (13, 17).

The limited information on the prominence and severity of hyperphagia in BBS, and limited assessment of the sensitivity and accuracy of tools used to detect it represent a critical barrier to the successful management of people with the disease, since effective treatments for hyperphagia and early-onset obesity (e.g., setmelanotide) may currently be underused (15, 19). To improve the management of hyperphagia in BBS, there is a need to develop and/or apply tools that are optimised for detecting and characterising hyperphagia, as currently existing clinical tools that are used to judge the degree of hyperphagia in rare MC4R pathway diseases may significantly underestimate the rate and severity of this life-limiting symptom (20).

This underestimation of hyperphagia could be due to patient-related factors (20) such as the disability paradox. The disability paradox is believed to reflect adaptations made by a person with a disability or their caregiver(s), whether practical (e.g., locking food cupboards), psychological (e.g., resilience of the patient to their disease (21)), or conceptual (e.g., lack of comparison to normal hunger/satiety cycles (16)). Other factors include shame or fear of stigmatisation (22), or intellectual/cognitive challenges sometimes associated with BBS (6, 23).

A lack of clinical tools, including the lack of BBS-validated tools to characterise hyperphagia-specific features of the disease, could also contribute to underestimation. Current scales have been validated only more generally for hyperphagia and/or in patients with other diseases, such as Prader–Willi syndrome (10, 24, 25). Another factor includes limitations in the fidelity of questionnaire data, which is a common tool used for symptom screening, including hyperphagia. For example, questionnaires ask participants to go from fact to subjective rating, but this conversion varies across participants, leading to significant noise.

Due to the challenges outlined above, methods are needed to capture the severity of hyperphagia in BBS more accurately. Semi-structured interviews provide an approach, complementary to questionnaires, for assessing clinical information. In contrast to questionnaires, semi-structured interviews focus directly on the realities of a person’s experience, allowing for direct comparisons between participants, especially when it comes to quantification. Semi-structured interviews acquire raw factual data, which the interviewer can compile and compare directly across participants. In addition, semi-structured interviews provide the opportunity to delve deeper and clarify information that might not be reflected accurately in the terminology in a questionnaire. Semi-structured interviews also allow the interviewer to identify (and resolve) inconsistencies, while probing beyond initial answers to capture information that may have been underreported in a questionnaire-only response due to the factors mentioned above.

In the current study, we conducted a cross-sectional, non-interventional assessment of hyperphagia severity in an adult population with BBS, using a standard, existing questionnaire, followed by a semi-structured, expert interview. In addition, we took advantage of a national BBS registry that facilitated inclusion of a broad and representative population.

## 2 Materials and methods

### 2.1 Study design, recruitment, and participants

We conducted a non-interventional, cross-sectional, mixed-method study. Eligible participants were adults (aged ≥18 years) with a clinical diagnosis of BBS, residing in the United Kingdom (UK) at the time of the study, and not currently or previously treated with setmelanotide. Participants were recruited from the BBS UK patient database, which identified adults with BBS who had previously given consent to be contacted for research purposes. A total of 51 participants completed the questionnaire. Ethics approval was obtained from the University of Portsmouth Research Ethics Committee prior to commencement of the study.

### 2.2 Inclusion and exclusion criteria for the full cohort

Participants were aged ≥18 years, had a clinical diagnosis of BBS, were able to provide consent, and possessed a sufficient level of English literacy to complete the questionnaire. Participants were excluded if they were currently prescribed or had previously taken setmelanotide.

**Figure 1:**
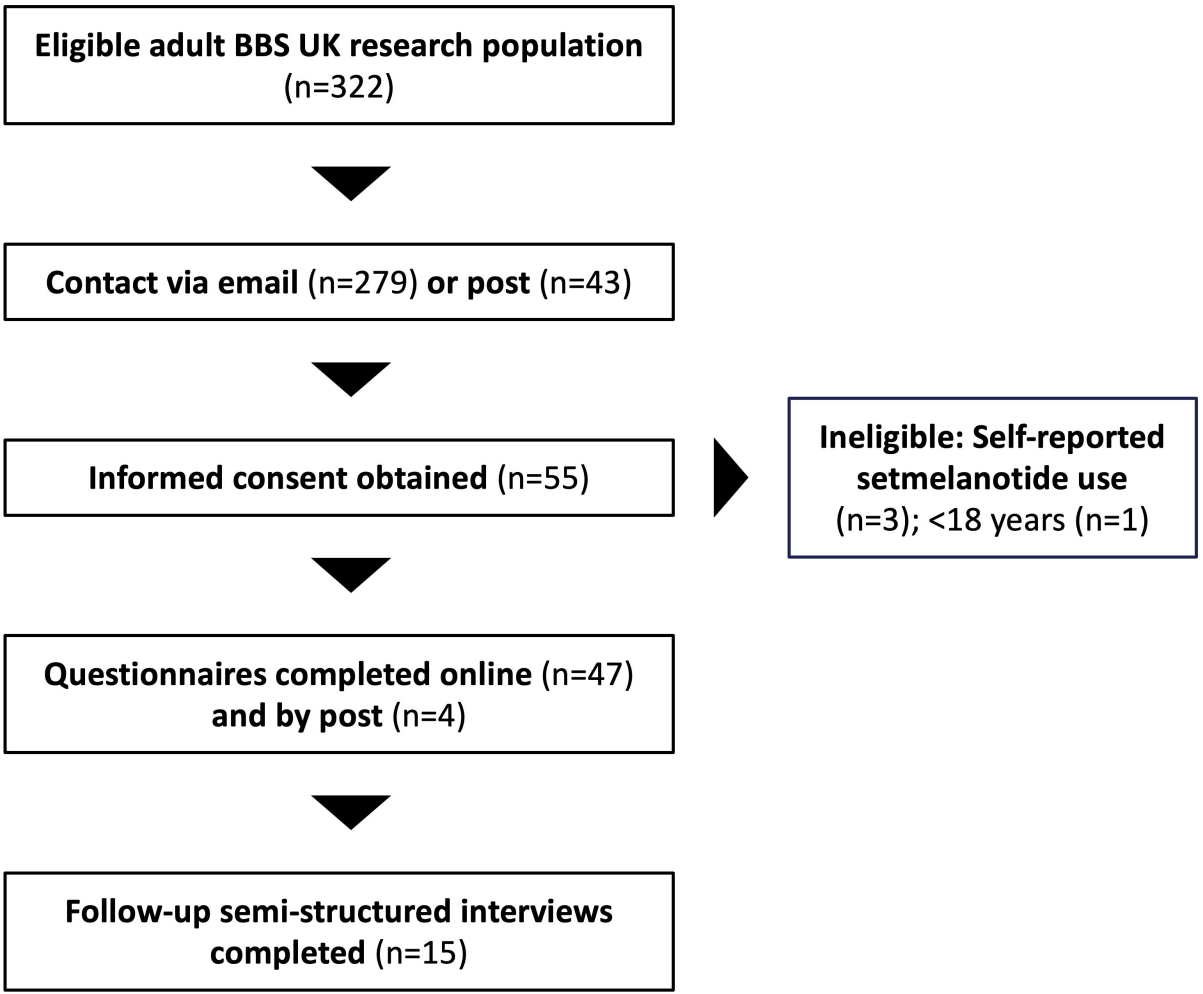
Recruitment flow chart. BBS, Bardet-Biedl syndrome.

The recruitment workflow is outlined in **Figure 1**. The registry identified 322 adults with BBS, and all were contacted by email or post to determine interest and eligibility. Informed consent to participate was obtained from 55 adults with BBS. Three participants were excluded for prior setmelanotide use, and one was excluded due to age <18 years. Study questionnaires were completed by 47 participants online and four by post. Fifteen of the participants who completed the questionnaires took part in semi-structured interviews.

### 2.3 Data collection

#### 2.3.1 Demographic information

We collected demographic information from each participant, including sex, race, ethnicity, and age (current age and age at diagnosis) (**Table 1**). Information on prior bariatric surgery, or concomitant or prior pharmacological treatments other than setmelanotide was not specifically collected as part of this study. Weight and height were self-reported and used to calculate body mass index (BMI).

**Table 1:** Demographics of adults with BBS.

Characteristics	Full cohort (N=51)	BMI ≥30 kg/m^2^ (n=39)	BMI <30 kg/m^2^ (n=12)
Sex, male, n (%)	25 (49.0)	16 (41.0)	9 (75.0)
Ethnicity, White, n (%)	42 (82.4)	32 (82.1)	10 (83.3)
Age at BBS diagnosis, years, mean (SD)	16.0 (11.5)	18.2 (13.1)	13.1 (8.1)
Age at time of study, years, n (%)			
18–24	16 (31.4)	12 (30.8)	4 (33.3)
25–34	13 (25.5)	10 (25.6)	3 (25.0)
35–44	10 (19.6)	7 (17.9)	3 (25.0)
45–54	4 (7.8)	4 (10.3)	0
55–64	6 (11.8)	5 (12.8)	1 (8.3)
≥65	1 (2.0)	0 (0.0)	1 (8.3)
Not specified	1 (2.0)	1 (2.6)	0
BMI at time of study, kg/m^2^, mean (SD)	36.5 (8.8)	40.0 (7.0)	25.4 (2.2)

BBS, Bardet-Biedl syndrome; BMI, body mass index; SD, standard deviation.

#### 2.3.2 Questionnaire

The 14-item hyperphagia questionnaire was completed online (n=47) or by post (n=4) and submitted anonymously. The questionnaire was designed to capture the behavioural, emotional, and functional aspects of hyperphagia (**Supplementary Table 1**). The first 11 questions in the questionnaire are adapted directly from the hyperphagia behavioural indicators (28). The remaining three questions were developed in consultation with BBS representatives and hyperphagia specialists to cover themes not appearing in Howell TA et al., 2023 (28). These questions were open-ended to elicit a more comprehensive response. A trained qualitative researcher scored the open-ended questionnaire responses and assigned them to specific categories, such as family intervention, external help sought, and self-perception, before aggregating them with multiple-choice question scores.

The final questionnaire score for each participant was calculated as the sum of all 14 items; the score for each question ranged from 1 to 4, allowing a maximum possible score of 56 for this evaluation. A score of 14 was considered no hyperphagia; 15 to ≤28 was considered mild hyperphagia; 29 to ≤42 was considered moderate hyperphagia; and 43 to ≤56 was considered severe hyperphagia (**Supplementary Table 2**). These predefined score thresholds enabled standardised categorisation of hyperphagia severity, facilitating descriptive comparison with interview-derived classifications.

#### 2.3.3 Semi-structured interview

Following completion of the questionnaire, participants were asked if they were interested in taking part in a semi-structured interview (**Supplementary Table 3**). Participation in the semi-structured interview component was voluntary and was based on participant willingness and availability. For those who chose to take part, the 45-minute follow-up semi-structured interviews were completed via Zoom or in person at the BBS UK Annual Conference in April 2025. The interviews were conducted by a trained qualitative researcher familiar with both BBS and hyperphagia, who explored the context behind questionnaire responses, including behavioural patterns, emotional experiences, caregiver interventions, and the overall impact of hyperphagia on daily functioning. Interviews were recorded and transcribed. The qualitative researcher coded the interview transcripts systematically and determined a severity score jointly with a BBS clinical expert. Severity scores were assigned using predefined descriptors aligned with published hyperphagia severity frameworks, incorporating behavioural, emotional, and functional aspects identified in the interview transcripts.

Additional scoring by a BBS clinical expert was included to identify contextual information and insights that may suggest underreporting, overreporting, or other nuances that were not captured by the questionnaire. Semi-structured interviews were intended to provide deeper contextual insights into patient-reported behaviours rather than a formal reference standard for hyperphagia severity.

Although both the questionnaire and semi-structured interview approach ultimately generated categorical hyperphagia severity classifications (mild, moderate, severe), the underlying basis for these classifications differed. Questionnaire-derived severity was based on a structured numerical scoring system with predefined cut-offs, whereas interview-derived severity was based on qualitative synthesis of contextualised patient-reported behaviours and experiences, interpreted by a trained qualitative researcher and reviewed with a BBS clinical expert. As such, the two approaches were designed to provide complementary perspectives on hyperphagia severity and are not strictly equivalent or directly interchangeable measures.

### 2.4 Data analysis

#### 2.4.1 Full cohort

Raw data for the full cohort were analysed using descriptive statistics (frequencies, proportions). No formal assessment of inter-rater reliability or reproducibility was conducted.

#### 2.4.2 BMI-based sub-analyses

Aligning with the population included in a previously published phase 3 clinical trial (19), a sub-analysis of adults with BBS and obesity (BMI ≥30 kg/m^2^) was performed to assess hyperphagia severity level in this population. Additionally, a sub-analysis for participants with BMI <30 kg/m^2^ was performed. These sub-analyses were exploratory and descriptive in nature and were conducted to contextualise findings within subgroups.

## 3 Results

### 3.1 Participants

In the full cohort, 51 adults with BBS (49.0% male, 82.4% White) completed the questionnaire, and 15 (41.0% male, 82.1% White) completed the semi-structured interview. Mean age (SD) at diagnosis of BBS was 16.0 (11.5) years, with a range from birth to 50 years. The mean BMI (SD) at time of study was 36.5 (8.8) kg/m^2^ (range 21.8–59.6) (**Table 1**).

For those who completed the semi-structured interviews, genotype information was available for 11 participants. Of these, 72.7% (8/11) had a variant in *BBS1*, 18.2% (2/11) *BBS2*, and 9.1% (1/11) *BBS5*. Overall, 73.3% (11/15) of interview participants had molecular confirmation of BBS, while all participants fulfilled the clinical diagnostic criteria for BBS.

Renal function data was available for 11 participants. This indicated that 18.2% (2/11) of interviewed participants had mild renal impairment (chronic kidney disease stage 2 or 3), and none had advanced renal disease, indicating that renal dysfunction is unlikely to have affected appetite or the interpretation of hyperphagia severity in this study.

The sub-analyses of participants with BMI ≥30 kg/m^2^ included 39 participants, with 13 of these also completing the semi-structured interview. Of those with BMI <30 kg/m^2^ (n=12), two completed the semi-structured interview. Demographics for both populations are shown in **Table 1**.

### 3.2 Hyperphagia severity distribution for questionnaire and semi-structured interview

The mean hyperphagia score from the questionnaire was 28.9 (out of a possible 56) for the full cohort (N=51); for participants with BMI ≥30 kg/m^2^ (n=39), the mean score for the questionnaire was 30.6. For the semi-structured interviews, the mean score was 3.6 out of 4 for the full cohort interview participants (n=15) and 3.7 for those with BMI ≥30 kg/m^2^ (n=13).

Percentages of participants with no, mild, moderate, or severe hyperphagia in the full cohort based on the questionnaire and on the semi-structured interviews are shown in **Table 2**; no participants were classified as having no hyperphagia.

**Table 2:** Hyperphagia severity level for the full cohort (N=51).

Severity level, n (%)	Questionnaire (N=51)	Semi-structured interview (n=15)
No hyperphagia	0 (0.0)	0 (0.0)
Mild hyperphagia	25 (49.0)	1 (6.7)
Moderate hyperphagia	23 (45.1)	4 (26.7)
Severe hyperphagia	3 (5.9)	10 (66.7)

Mild hyperphagia was classified in 49.0% of questionnaires versus 6.7% of interview participants; moderate in 45.1% versus 26.7%, respectively; and severe in 5.9% versus 66.7%, respectively.

All interview participants with available genotype data (11/15), two were classified as having moderate hyperphagia, with nine classified as severe hyperphagia based on semi-structured interviews. Notably, individuals with *BBS1* carrying the common *M390R* homozygous variant – typically associated with a comparatively milder BBS phenotype in some clinical manifestations – also demonstrated severe hyperphagia. Percentages of participants with no, mild, moderate, and severe hyperphagia in the sub-analysis of participants with BMI ≥30 kg/m^2^ based on the questionnaire and semi-structured interviews are shown in **Table 3**; in this sub-analysis, no participants were classified as having no hyperphagia. Mild hyperphagia was classified in 41.0% of questionnaires versus 0.0% of interview participants; moderate in 51.3% versus 30.8%, respectively; and severe in 7.7% versus 69.2%, respectively.

**Table 3:** Sub-analysis for participants with BBS and BMI ≥30 kg/m^2^ (n=39) and <30 kg/m^2^ (n=12).

Severity level, n (%)	BMI ≥30 kg/m^2^		BMI <30 kg/m^2^	
	Questionnaire (n=39)	Semi-structured interview (n=13)	Questionnaire (n=12)	Semi-structured interview (n=2)
No hyperphagia	0 (0.0)	0 (0.0)	0 (0.0)	0 (0.0)
Mild hyperphagia	16 (41.0)	0 (0.0)	9 (75.0)	1 (50.0)
Moderate hyperphagia	20 (51.3)	4 (30.8)	3 (25.0)	1 (50.0)
Severe hyperphagia	3 (7.7)	9 (69.2)	0 (0.0)	0 (0.0)

BBS, Bardet-Biedl syndrome; BMI, body mass index.

In participants without obesity (BMI <30 kg/m^2^), only mild (75.0%) or moderate hyperphagia (25.0%) was scored. The two participants who participated in semi-structured interviews were classified as having moderate or severe hyperphagia (**Table 3**). Notably, none of the adults with BBS without obesity scored themselves as having severe hyperphagia, compared with three adults with BBS and BMI ≥30 kg/m^2^.

#### 3.2.1 Comparison of questionnaire and semi-structured interview scores at individual participant level

**Figure 2:**
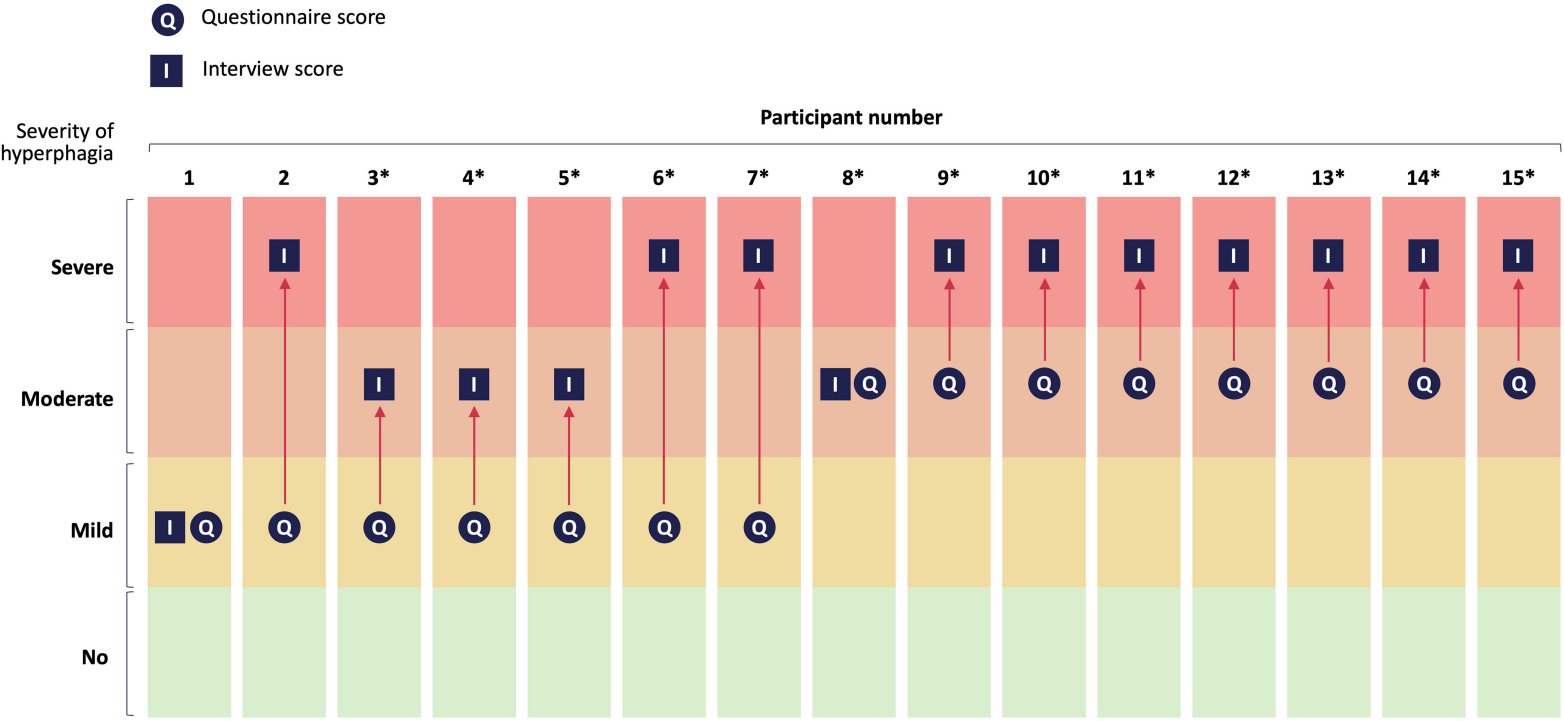
Difference in severity level of hyperphagia between self-reported questionnaire and semi-structured interview scores. Maximum score 56, with ranges: 14 considered no hyperphagia; 15 to ≤28 considered mild hyperphagia; 29 to ≤42 considered moderate hyperphagia; 43 to ≤56 considered severe hyperphagia. *Participants #3–#15 had a BMI ≥30 kg/m^2^. BMI, body mass index.

The difference in hyperphagia severity levels among the 15 participants from the full cohort who completed both the questionnaire and semi-structured interview is reported in **Figure 2**.

Of the 15 participants who completed the interview, none had self-reported a severe level of hyperphagia in the questionnaire; eight reported answers corresponding to moderate hyperphagia, and seven reported answers corresponding to mild hyperphagia. Almost all interview participants (n=13, 86.7%) shifted to a higher severity level with the semi-structured interview, compared with the questionnaire; only two participants had identical severity classifications across both methods.

In the sub-analysis of participants with BMI ≥30 kg/m^2^, of the 13 participants who completed the interview, none had reported a severe level of hyperphagia in the questionnaire; eight self-reported answers corresponding to moderate hyperphagia, and five self-reported answers corresponding to mild hyperphagia. Almost all interview participants (n=12, 92.3%) shifted to a higher severity level with the semi-structured interview, compared with the questionnaire.

**Table 4** provides a selection of representative examples of differences between questionnaire answers and reported responses in semi-structured interviews.

**Table 4:** Representative examples of differences in responses between questionnaire and semi-structured interview.

Participant	Question	Questionnaire	Interview
Participant A	How distressed or upset do you feel when denied food?	Not at all	Participant A struggles with constant thoughts about food throughout the day; experiences frustration when feeling hungry shortly after eating.
	How soon do you feel hungry again after eating a normally sized meal?	At the next mealtime	Participant A feels miserable and agitated when unable to eat when hungry. Experiences anxiety and self-consciousness about eating habits due to comments from others.
	Overall hyperphagia severity score	Moderate	Severe
Participant B	How many meals and snacks do you typically eat each day?	3 meals, 1–2 snacks	Participant B snacks throughout the day to manage hunger between meals.
	How distressed or upset do you feel when denied food?	Not at all	Participant B feels annoyed about having to eat again so soon after a meal.
	Overall hyperphagia severity score	Mild	Severe
Participant C	How many meals and snacks do you typically eat each day?	3 meals, 1–2 snacks	Participant C experiences frequent hunger and food cravings, even shortly after eating. Struggles with eating out of boredom rather than actual hunger. Finds it difficult to resist snacks and treats when they are available. Experiences guilt and frustration after overeating or eating at inappropriate times. Struggles with the side effects of steroid medication, which increase his appetite. Struggles with hunger/portions; sometimes sneaks food.
	How distressed or upset do you feel when denied food?	Mildly	Participant C experiences sadness when unable to eat when hungry. Frustration with the cycle of eating, weight gain, and steroid medication. Happiness when eating to satisfy cravings, but followed by guilt.
	Overall hyperphagia severity score	Mild	Severe
Participant D	How many meals and snacks do you typically eat each day?	3 meals, 1–2 snacks	Constant thoughts about food (70% of the time) distract from daily tasks.
	How much do hunger and eating behaviours interfere with your ability to perform daily activities (self-care, getting around, leisure activities, and work or school)?	Not at all	They often have to stop housework to eat, sometimes forgetting to return to the task. Overeating causes physical discomfort, making it hard for them to move and complete tasks. Keeps snacks in their room that only their mother knows about. Feels sad, guilty, and upset about their constant thoughts of food. Feels depressed and left out due to their weight and inability to do certain activities.
	Overall hyperphagia severity score	Moderate	Severe

Hyperphagia severity score: red box = severe; orange box = moderate; yellow box = mild.

## 4 Discussion

In the full cohort analysis, semi-structured interviews revealed a substantially higher proportion of severe hyperphagia (66.7%), compared with self-reported questionnaires (5.9%). This resulted in most (86.7%) participants being reclassified to a higher severity level through interviews. The magnitude of discrepancy between methods suggests that reliance on questionnaire-based assessments alone may substantially underestimate the clinical burden of hyperphagia in adults with BBS.

In the sub-analysis of participants with BMI ≥30 kg/m², 69.2% were classified as having severe hyperphagia by semi-structured interviews versus only 7.7% by questionnaire, again demonstrating substantial underreporting of hyperphagia severity when relying solely on self-assessment tools. The BMI-based sub-analysis was exploratory and descriptive in nature. Accordingly, these results should be interpreted as supportive rather than central to the study conclusions.

These findings highlight the limitations of questionnaire-based approaches and the potential value of incorporating semi-structured interviews as a complementary approach to capture aspects of hyperphagia that may be underreported in questionnaires Importantly, all participants, including those with BMI ≤30 kg/m^2^, were classified as having hyperphagia.

Our findings demonstrate systematic underreporting of hyperphagia in self-reported questionnaires, with semi-structured interviews consistently capturing higher severity levels and revealing behaviours not disclosed through questionnaire-based instruments. This is consistent with the hypothesis that factors such as the disability paradox, shame/stigmatisation, and cognitive limitations may prevent accurate questionnaire self-assessment in adults with BBS (6, 10, 16, 21–26). For example, we noted discrepancies in the reporting of hyperphagic behaviours (e.g., secretive eating, binge episodes, caregiver intervention, and emotional dysregulation): these behaviours were more widely disclosed during interviews than in questionnaires. In interviews, it also became apparent that participants normalised their behaviours as part of their lifelong experience, often not recognising their eating patterns and behaviours as abnormal. The importance of using a hybrid assessment approach, combining interviews and questionnaires, has been discussed previously in other domains, such as traumatic brain injury (27). Our findings support, alongside questionnaires, the importance of interviews including subjective scoring, and consensus scoring in accurately characterising hyperphagia.

As the multiple-choice questions were based on previous literature, the current study included nocturnal eating in severity indicator measures (28). Nocturnal eating appears to be an important feature in some other diseases that lead to hyperphagia, including Prader–Willi syndrome (25). However, nocturnal eating was not reported frequently in this population of adults with BBS. Of the questionnaire respondents, 45 out of 51 reported no nocturnal eating, and none of the 15 interview participants reported this behaviour in the semi-structured interviews. The low occurence of nocturnal eating in the current study may have been driven by the disability paradox and the fact that people living with BBS typically experience early-onset, progressive nighttime blindness (nyctalopia), which may discourage food-seeking behaviours after dark. This study shows that disease-specific tailored hyperphagia questionnaires are needed, and that confounding/complementary features of a given patient’s responses, such as nyctalopia, should be considered when interpreting variance across patient symptom spectrums.

This study had several limitations. First, questionnaire- and interview-derived severity scores were not generated using a validated, BBS-specific instrument, and no formal assessment of inter-rater reliability, reproducibility, or scoring standardisation was performed. As such, variability in interpretation cannot be excluded. Second, only a subset of participants (n=15/51) completed semi-structured interviews, introducing the potential for selection bias. Participation in the interview component was voluntary, and characteristics of individuals opting to participate may differ from those who declined (e.g., symptom awareness, engagement, or disease burden). Accordingly, the interview findings should be interpreted with appropriate consideration of potential selection bias; however, the consistent magnitude and direction of the differences observed support a robust signal of underestimation in questionnaire-based assessments. Third, interviews inherently involve probing and contextual exploration, which may increase the likelihood of hyperphagia symptom disclosure and confirmation bias. This may reflect more comprehensive capture of behaviours not reported in self-administered questionnaires, highlighting differences in data capture between methods rather than solely indicating inflation of symptom severity. In addition, differences in how severity was operationalised between the questionnaire (structured score-based categorisation) and interviews (interpretive synthesis of contextual data) limit direct comparability between methods. Fourth, this study did not directly collect data on prior bariatric surgery, or concomitant or prior pharmacological treatments (e.g., glucagon-like peptide-1 receptor agonists), which may have influenced appetite and related behaviours, and their potential influence on hyperphagia severity cannot be excluded. Finally, as BBS is a rare disease, and this was a single-country study, the sample size was relatively small and may not be generalisable to the wider BBS population. While the relative size is representative of the overall population characteristics, larger studies are needed to replicate and validate our findings and support the development of standardised, validated hyperphagia assessment tools in BBS.

Taken together, our findings highlight potential limitations of questionnaire-based assessments and support further investigation into optimised, standardised approaches for evaluating hyperphagia severity in BBS. The combination of structured and qualitative approaches may provide complementary insights into hyperphagia experience and severity that may not be captured by a single tool alone.

## 5 Conclusions

Our findings from semi-structured interviews suggest a high prevalence of severe hyperphagia in adults with BBS in the UK, however reliance on self-reported questionnaires alone may lead to underreporting of hyperphagia severity. To enhance the clinical understanding of hyperphagia through complementary perspectives, we recommend incorporating semi-structured interviews reviewed by a BBS expert, which may improve both the diagnosis and management of hyperphagia in adults with BBS. Further research is needed to develop standardised, reliable, and validated hyperphagia assessment tools tailored to people living with BBS. In parallel, greater awareness among healthcare professionals is needed regarding hyperphagia and its variable presentation across rare MC4R pathway diseases, such as BBS, as well as the strengths and limitations of existing assessment tools and the role of alternative approaches, such as interviews, in supporting accurate diagnosis and treatment.

## Data Availability

All data generated or analysed for this study are included in this article and its **Supplementary Material** files. Further enquiries can be directed to the corresponding author.
