# Supplementary material for: Hyperphagia severity is underestimated in adults with Bardet-Biedl syndrome – a mixed-method cross-sectional study in the United Kingdom
Source: Front Endocrinol (Lausanne). 2026 Jul 2;17:1858350. doi: 10.3389/fendo.2026.1858350 (PMC13373750; doi:10.3389/fendo.2026.1858350)
Supplement: Supplementary file 1 [file DataSheet1.pdf]

Supplementary Information

Supplementary Table 1. Hyperphagia severity questionnaire

Screening form

Have you been diagnosed with Bardet-Biedl Syndrome (BBS)?

- Yes
- No

Are you taking or have you previously taken a medication called setmelanotide (a daily injection that helps with hunger and weight loss)?

- Yes
- No

Demographics

Age:

- 18–24
- 25–34
- 35–44
- 45–54
- 55–64
- 65 and over

Gender:

- Man
- Woman
- Non-binary
- I prefer not to say
- I prefer to self-describe: _____________

Age at BBS diagnosis: __________

Your current weight (in kilograms): _____________

Your height (in feet and inches): _____________

Do you consider yourself:

- Obese
- Overweight
- A few extra pounds
- Normal weight (healthy BMI)
- Below normal weight

Ethnicity:

- South Asian
- East Asian
- Black, Caribbean, or African
- Mixed or multiple ethnic groups
- White
- Other ethnic group

What is your current living situation?

- Living alone

Living with parents/guardians
Living with spouse/partner
Living with roommates/housemates
Living in a residential care home
Other: _____________

Who mostly makes your meals?

Self
Parents/guardians
Spouse/partner
A caregiver
Other: _____________

Who mostly does the grocery shopping in your household?

Self
Parents/guardians
Spouse/partner
A caregiver
Other: _____________

Subjective experience

1. How often do you feel full after eating a normally-sized meal?
   - 1: Always
   - 2: Sometimes
   - 3: Rarely
   - 4: Almost never
2. How soon do you feel hungry again after eating a normally-sized meal?
   - 1: At the next mealtime
   - 2: Within 2 hours
   - 3: Within 1 hour
   - 4: Almost immediately
3. How often does thinking about food interfere with your normal activities of daily living?
   - 1: Never
   - 2: Sometimes
   - 3: Often
   - 4: Almost always

Observable behaviours

4. How often do you overeat to the point of discomfort?
   - 1: Rarely
   - 2: Sometimes
   - 3: Often
   - 4: At most meals
5. How many meals and snacks do you typically eat each day?
   - 1: 3 meals and 1–2 snacks
   - 2: 3 meals and more than 2 snacks
   - 3: More than 3 meals and more than 3 snacks
   - 4: Almost constant eating
6. How often do you eat during the hour before going to bed?

    - 1: Rarely
    - 2: Sometimes
    - 3: Often
    - 4: Almost every night
7. How often do you eat when waking up during the night?
    - 1: Never
    - 2: About once per week
    - 3: 2–3 times per week
    - 4: Almost every night
8. How often do you try to eat food without others knowing?
    - 1: Rarely
    - 2: Occasionally
    - 3: About twice per week
    - 4: Almost every day

Impact

9. How distressed or upset do you feel when denied food?
    - 1: Not at all
    - 2: Mildly
    - 3: Moderately
    - 4: Extremely
10. How much do hunger and eating behaviours interfere with your ability to perform daily activities (self-care, getting around, leisure activities, and work or school)?
    - 1: Not at all
    - 2: Slightly
    - 3: Moderately
    - 4: Severely
11. How much do hunger and eating behaviours interfere with your relationships with family and friends?
    - 1: My hunger and eating behaviours do not interfere with my relationships with family and friends
    - 2: My hunger and eating behaviours cause slight problems in my relationships with family and friends
    - 3: My hunger and eating behaviours cause moderate problems in my relationships with family and friends
    - 4: My hunger and eating behaviours cause severe problems in my relationships with family and friends

---

Qualitative questions

12. Do your family, friends, or caregivers help you manage your eating habits? If yes, describe how they help or intervene. (300-word limit)

    Enter answer here ________________
13. Have you ever sought treatment or help managing your eating habits? If yes, what have you tried (e.g., therapy, medication, dietary changes), and did it help? (300-word limit)

    Enter answer here ________________
14. In your own words, can you describe what it is like for you to manage your hunger? (300-word limit)

    Enter answer here ________________

Supplementary Table 2. Hyperphagia severity scoring table

Theme	#	No hyperphagia (1)	Mild hyperphagia (2)	Moderate hyperphagia (3)	Severe hyperphagia (4)
Multiple choice questions (#1–11)					
**Subjective experience**	1	You usually eat a normally sized meal and feel full	You sometimes do not feel full after a normally sized meal	You usually do not feel full after a normally sized meal	You almost never feel full after a normally sized meal
	2	You can eat a normal meal and not feel hungry until the next meal	You become hungry again within 2 hours after eating a meal	You become hungry again within 1 hour after eating a meal	You become hungry again almost immediately after eating a meal
	3	Thinking about food does not interfere with your normal activities of daily living	Thinking about food sometimes interferes with your normal activities of daily living	Thinking about food often interferes with your normal activities of daily living	Thinking about food almost always interferes with your normal activities of daily living
**Observable behaviours**	4	You rarely overeat to the point of discomfort	You sometimes overeat to the point of discomfort	You often overeat to the point of discomfort	You overeat to the point of discomfort at most meals
	5	You eat 3 meals per day with 1 or 2 snacks	You eat 3 meals per day with more than 2 snacks	You eat more than 3 meals per day with more than 3 snacks	You eat almost constantly
	6	You usually do not eat during the hour before you go to bed	You sometimes eat during the hour before you go to bed	You often eat during the hour before you go to bed	You eat during the hour before you go to bed almost every night
	7	You do not eat when you wake up during the night	You eat when you wake up during the night about once per week	You eat a large number of calories when you wake up during the night about 2–3 times per week	You eat a large number of calories when you wake up during the night almost every night
	8	You rarely try to sneak food without people knowing	You occasionally try to sneak food without people knowing	You try to sneak food without people knowing about twice per week	You try to sneak food without people knowing almost every day
**Impact**	9	You do not become overly distressed or upset when denied food	You become mildly distressed or upset when denied food	You become moderately distressed or upset when denied food	You become extremely distressed or upset when denied food
	10	Hunger and eating behaviour do not interfere with your ability to perform daily activities such as self-care, getting around, leisure activities, and work or school	Because of hunger and eating behaviour, you have slight problems performing daily activities such as self-care, getting around, leisure activities, and work or school	Because of hunger and eating behaviour, you have moderate problems performing daily activities such as self-care, getting around, leisure activities, and work or school	Because of hunger and eating behaviour, you have severe problems performing daily activities such as self-care, getting around, leisure activities, and work or school
	11	Hunger and eating behaviour do not interfere with your relationships with family and friends	Because of hunger and eating behaviour, you have slight problems with your relationships with family and friends	Because of hunger and eating behaviour, you have moderate problems with your relationships with family and friends	Because of hunger and eating behaviour, you have severe problems with your relationships with family and friends
Open-ended questions (#12–14)					

**Family intervention**	12	No caregiver intervention required	Requires minimal intervention, such as reminders to eat balanced meals	Caregivers/family take active steps to prevent overeating, such as monitoring meals, limiting portions, or restricting certain foods	Extreme measures are required to control food access, such as locking food away, 24/7 supervision, or restricting all access to certain foods
**External help**	13	No need for treatment; participant feels their eating habits are under control	Has considered or tried some form of support (general diet advice, self-help), but does not feel an urgent need for treatment	Has actively sought help for difficulty managing hunger through structured methods (therapy, medication, clinician-supervised diet plans)	Has tried multiple treatments without success OR has required medical intervention due to the severity of food-seeking behaviours
**Self-perception**	14	Hunger levels are normal and do not interfere with their daily life	Experiences some difficulty managing hunger, but it is manageable	Struggles frequently with hunger or food preoccupation	Constant, distressing hunger that dominates their thoughts and behaviours

Supplementary Table 3. Semi-structured interview questions

Theme 1: Subjective experience

- Feeling of fullness and hunger cycles:
    - Describe how you feel after eating a typical meal. Do you often feel satisfied or still hungry?
    - How soon after eating a meal do you start to feel hungry again, and how does this affect your day?
- Food preoccupation:
    - How often do you find yourself thinking about food? Would you say it distracts you from daily tasks or activities?
    - When you think about food, what thoughts or feelings arise for you (e.g., anticipation, stress, frustration)?

Theme 2: Observable behaviours

- Eating patterns:
    - Walk me through a typical day of meals and snacks. How does your eating routine differ from day to day?
    - Do you notice times when you feel more inclined to eat (e.g., before bed, waking up during the night)? If so, what do you think triggers this?
- Hidden behaviours:
    - Some people with hyperphagia describe sneaking food or eating in secret. Is this something you do or have done? If so, how does it affect your daily life or interactions with others?
    - Have there been moments when you felt you ate beyond what you intended? If yes, how do you feel after those instances?

Theme 3: Impact on daily life

- Emotional impact:
    - How do you usually feel if you're unable to eat when you're hungry? Does this lead to difficult emotions or behaviours?
    - Have your feelings about food ever caused distress or frustration in your day-to-day life? Please describe a specific example.
- Interference with daily activities:
    - How have your eating habits ever interfered with your ability to do things like work, school, or hobbies?
    - How has your hunger or eating behaviours affected your ability to care for yourself (e.g., hygiene, mobility, errands)?
- Relationships:
    - How do your eating habits impact your relationships with family or friends?
    - Have your loved ones ever expressed concerns about your eating habits? How do you respond to their concerns?
